# Modulation of immune cell function, IDO expression and kynurenine production by the quorum sensor 2-heptyl-3-hydroxy-4-quinolone (PQS)

**DOI:** 10.3389/fimmu.2022.1001956

**Published:** 2022-10-28

**Authors:** Joy Ogbechi, Yi-Shu Huang, Felix I. L. Clanchy, Eirini Pantazi, Louise M. Topping, L. Gail Darlington, Richard O. Williams, Trevor W. Stone

**Affiliations:** ^1^ The Kennedy Institute of Rheumatology, Nuffield Department of Orthopaedics, Rheumatology and Musculo-skeletal Sciences (NDORMS), University of Oxford, Oxford, United Kingdom; ^2^ Internal Medicine, Ashtead Hospital, Ashtead, United Kingdom

**Keywords:** arthritis (including rheumatoid arthritis), PQS signaling, Indoleamine 2 3-dioxygenase (IDO), kynurenine (KYN), tolerance, regulatory T (Treg) cells, Th17 cells and Treg cells, quorum sensing (QS)

## Abstract

Many invasive micro-organisms produce ‘quorum sensor’ molecules which regulate colony expansion and may modulate host immune responses. We have examined the ability of *Pseudomonas* Quorum Sensor (PQS) to influence cytokine expression under conditions of inflammatory stress. The administration of PQS *in vivo* to mice with collagen-induced arthritis (CIA) increased the severity of disease. Blood and inflamed paws from treated mice had fewer regulatory T cells (Tregs) but normal numbers of Th17 cells. However, PQS (1μM) treatment of antigen-stimulated lymph node cells from collagen-immunised mice *in vitro* inhibited the differentiation of CD4+IFNγ^+^ cells, with less effect on CD4+IL-17+ cells and no change in CD4+FoxP3^+^Tregs. PQS also inhibited T cell activation by anti-CD3/anti-CD28 antibodies. PQS reduced murine macrophage polarisation and inhibited expression of IL1B and IL6 genes in murine macrophages and human THP-1 cells. In human monocyte-derived macrophages, IDO1 gene, protein and enzyme activity were all inhibited by exposure to PQS. TNF gene expression was inhibited in THP-1 cells but not murine macrophages, while LPS-induced TNF protein release was increased by high PQS concentrations. PQS is known to have iron scavenging activity and its suppression of cytokine release was abrogated by iron supplementation. Unexpectedly, PQS decreased the expression of indoleamine-2, 3-dioxygenase genes (IDO1 and IDO2), IDO1 protein expression and enzyme activity in mouse and human macrophages. This is consistent with evidence that IDO1 inhibition or deletion exacerbates arthritis, while kynurenine reduces its severity. It is suggested that the inhibition of IDO1 and cytokine expression may contribute to the quorum sensor and invasive actions of PQS.

## Introduction

Many pathogenic bacteria and other invasive micro-organisms can produce an immunosuppressive local environment in the host which would permit or encourage their own survival and development. In many cases this is achieved by a system of quorum sensing (QS) by which the invading microbes secrete signalling molecules to modulate colony size and density (1–7). When the invading colony size is small, the ambient concentrations of QS Signalling Molecules (QSSMs) are maintained at low levels to restrict proliferation, growth and migration, minimising any activation of the host immune system. As colony density rises to a critical threshold (the ‘quorum’) which should be sufficient to overcome host defences, the relationship between QSSM concentration and receptor activity reverses to yield a positive feedback system in which the QSSMs promote proliferation and increased migration. The QSSMs also regulate the generation of virulence factors which are injurious to host tissues and which facilitate microbial invasion and dissipation (8–11). Virulence factors include a wide range of molecules which can include proteases (12–14) some of which may contribute to the induction of host immune tolerance by the induction of indoleamine-2,3-dioxygenase-1 (IDO1) (14). This is accompanied by synchronised mitosis and expression of genes involved in self-protection, such as adhesion molecules CD11b (15), and a more aggressive inhibition of host immune defences. Together, the suppression of host immunity, promotion of pathogen survival, and the generation of bacterial biofilms within tissues and on foreign surfaces (e.g. catheters), produce conditions which are extremely stable and lead to bacterial pockets which are highly resistant to antibiotic treatments and with local QSSM concentrations of over 100 μM (16, 17). It is therefore essential to appreciate the sites and mechanisms of action of QSSMs in the invading bacterial and host tissues in the development of novel therapies.

The current investigation centres on the QS system of 2-alkyl-4-quinolones (2A4Q) represented by 2-heptyl-3-hydroxy-4-quinolone, more commonly referred to as the *Pseudomonas* Quorum Sensor (PQS) (or *Pseudomonas* Quinolone System) from its original identification in the major human pathogenic commensal *P. aeruginosa* although it is also expressed by many other bacteria (1). It has been reported that PQS exhibits anti-inflammatory activity by suppressing cytokine generation and inhibiting the activation of NFκB (18, 19) while the promotion of IFN-γ related genes inhibits PQS activity and enhances host resistance (20–22). The switch to high pathogenicity and the generation of virulence factors can also induce host immune mechanisms to attack damaged host cells – in effect triggering autoimmunity by a positive feedback of increased bacterial growth and a re-focussing of host immunity on self (2, 23, 24).

The situation has been compared with the state of sepsis, in which QSSMs may be involved (25). The present study was designed to assess whether PQS would affect cytokine expression under conditions of inflammatory challenge as seen in models of arthritis and colitis. In addition, an *in vitro* analysis compared changes in T cell populations and cytokine generation in cells taken from normal control animals and those with arthritis to determine the balance of pro-inflammatory and anti-inflammatory activity. This includes the ratio of Th17 cells to regulatory T cells (Tregs) which is now regarded as pivotal in many autoimmune conditions and cancers (26) and an examination of the tolerogenic enzyme IDO1 (27).

## Materials and methods

### Ethical statement

Human apheresis cones were obtained with informed consent from the National Health Service Blood Service (REC: 11/H0711/7). All procedures were approved by the Animal Welfare Ethical Review Board and were undertaken in accordance with personal and project licences issued by the UK Home Office under the UK Animals (Scientific Procedures) Act, 1986.

### Collagen-induced arthritis (CIA)

Details of the model have been published previously (28). Briefly, male DBA/1 mice were immunised subcutaneously with 200 μg of bovine type II collagen emulsified in Complete Freund’s Adjuvant (CFA) (BD Biosciences) at the base of the tail and on the flank. After immunization, the mice were monitored daily for symptoms of arthritis. Once an animal showed signs of arthritis, it was randomly assigned to a treatment or control group and monitored daily. Animals received PQS (10 mg/kg per day, i. p.) or vehicle and were treated until day 10. The development of arthritic symptoms and their severity was scored by an experienced, blinded investigator as follows: 0 = normal, 1 = slight swelling and/or erythema, 2 = pronounced swelling, and 3 = ankylosis. All four limbs were scored, giving a maximum possible score of 12 per animal. On day 10 the animals were euthanised and the paws, inguinal lymph nodes, spleen and blood were collected and single-cell suspensions were obtained and analysed by flow cytometry.

### Antigen-induced arthritis (AIA)

Male and female C57BL/6 mice, 8-12 weeks of age, were immunised subcutaneously with 100 μg of methylated bovine serum albumin (mBSA) emulsified with an equal volume of CFA. On day 21, 100 μg of mBSA was administered by intra-articular injection into the right knee joint while the left knee joint received PBS, as a control. Changes in knee swelling were determined by comparing to measurements made prior to the intra-articular injection. Mice were treated with PQS (10mg/kg) or vehicle during either the post-immunisation, pre-intra-articular injection period or after the intra-articular injection period. After 7 days the animals were euthanised, inguinal lymph nodes and knee joints were collected and single-cell suspensions were obtained and analysed by flow cytometry.

### Weight-bearing

Knee joint nociception was evaluated using a dynamic weight bearing apparatus (Bioseb, France) as previously described (29). For testing, the mouse was placed in the device and allowed to move freely for a period of 4 minutes. Integrated analysis of video and pressure sensors by the software determined the weight distribution of each of the four paws.

### Dextran Sulphate-induced colitis (DSS colitis)

Dextran sodium sulphate (DSS; MP Biomedicals, Cat No 160110 (MW: 36,000-50,000) was administered in the drinking water (3%) for 5 days (30). Body weight and appearance were monitored daily and PQS was administered intraperitoneally daily. Mice were subsequently euthanized 7 d after the start of DSS administration or within 24 h if they exhibited a weight loss of more than 15% of their initial body weight. On removing the colon, its length was measured and a portion of distal colon and caecum were fixed in neutral buffered 10% formalin (CellPath, ref BAF-6000-08A) followed by paraffin embedding. The fixed tissue was stained with eosin and haematoxylin. Images were produced from at least five sections per organ using an Axioscan-Z1 platform (Zeiss) with Zen-2.3 software and using an Olympus BX51 microscope. Slides were scored blindly using 3 sections per sample as described previously (31).

### Mouse T cell preparation

Single cell suspensions were prepared from the lymph node and spleen of C57BL/6 mice and CD4^+^ T cells were isolated using the CD4^+^ T Cell Isolation Kit (130-104-454, Miltenyi Biotec). Cells were cultured in RPMI medium supplemented with 10% FBS, 50 μM 2-mercaptoethanol and 10,000 U/ml penicillin/streptomycin. Cells were activated with plate-bound anti-mouse CD3 (5 μg/ml; clone 145-2C11), and soluble anti-mouse CD28 (2 μg/ml; clone 37.51, eBioscience) in RPMI for four days in the following differentiation media:-

(a) for Th1 - 10 ng/ml IL-12 (200–12), 10 ng/ml IL-2, 5 µg/ml anti-IL-4 (504122, BioLegend);

(b) for Th17 - 50 ng/ml IL-6 (216–16), 10 ng/ml IL-1β (211-11B), 10 ng/ml IL-23 (200–23), 5 ng/ml TGF-β (100–21), 5 µg/ml anti-IL-4, 5 µg/ml anti-IFN-γ (505834, BioLegend);

(c) for Tregs - 10 ng/mL IL-2 (200-02, PeproTech) and 10 ng/mL TGF-β DMSO or the indicated concentration of PQS was added from day 1 of stimulation and cell populations were analysed by FACS after 4 days

### Murine bone marrow-derived macrophages (BMDMs)

Bone marrow cells were harvested from the femurs of mice. To derive naïve (M0) macrophages, bone marrow cells were cultured in complete RPMI 1640 with 50 ng/ml M-CSF (315-02, PeproTech) for 7 days, of which 5 ml were replenished by new complete RPMI 1640 with 50 ng/ml M-CSF at day 3. For M1 and M2 differentation, M0 cells were then treated with LPS (L2880, Sigma-Aldrich) and/or IFNγ (315-05, PeproTech) for M1 and IL-4 (214-04, PeproTech) and/or IL-10 (210-10, PeproTech) for M2. To investigate the effect of PQS on the differentiation of M1 and M2, PQS (0.05 to 20 μM) was co-treated with the cytokines known to promote M1 and M2 differentiation. After overnight culture, cells were lysed for gene expression by qPCR.

### Human monocyte-derived macrophages

Human apheresis cones were obtained with informed consent from the National Health Service Blood Service (REC: 11/H0711/7). Peripheral Blood Mononuclear Cells (PBMCs) were isolated as previously described (32) using density separation (Lympholyte^®^, Cedarlane). Monocytes were isolated by positive immunomagnetic selection (Miltenyi) according to the manufacturer’s instructions (33). Monocytes (10^6^ per mL) were cultured in 10 cm dishes for up to 7 days in complete RPMI (10% FBS, 1% penicillin/streptomycin) supplemented with 50 ng/mL of human M-CSF (300-25, PeproTech) to generate monocyte derived macrophages (MDMs).

### THP-1-derived macrophages

The THP-1 cell line is a commonly used surrogate for macrophage studies, being derived from human leukaemia cells (34, 35). THP-1-derived M0, M1 and M2 macrophages were differentiated and treated with PQS by the same protocols as mentioned above in the section of murine bone marrow-derived macrophages

### Human Th17 differentiation

Naïve T cells were isolated from human PBMCs by magnetic activated cell sorting using the Naïve human CD4^+^ T cells Isolation Kit II, (Miltenyi, 130-094-131). *In vitro* stimulation was performed with plate-bound anti-CD3 antibody at 0.5 μg/ml (317315) (clone OKT3; BioLegend, LEAF grade) and 2 μg/ml anti-CD28 antibody (302923) (clone CD28.2; BioLegend, LEAF grade) in 200 μl RPMI at 2x10^5^ cells per well. Recombinant cytokines (PeproTech) were added at the following concentrations: IL-6 (200–06), 50 ng/ml; IL-1β (200–01) and IL-23 (200–23), 10 ng/ml and TGF-β (200–21)1 ng/ml, and neutralizing antibodies against IFN-γ (502404) and IL-4 (500707) were used at 2 μg/ml (BioLegend, LEAF grade). Cells were split 1 in 4 on day 3 and 200 μl of fresh complete RPMI containing cytokines and antibodies were added to the wells. This replenishment was repeated on day six. On day 7 cells were stimulated for 5 h with 500 ng/ml PMA (Sigma-Aldrich) and 1 mg/ml ionomycin (Sigma-Aldrich) in the presence of Brefeldin A (Sigma-Aldrich). Cells were stained with an antibody against CD4 (45-0049-42, eBioscience) as well as a Fixable Viability Dye (BioLegend) then fixed and permeabilized using the FoxP3 Staining Buffer Set (eBioscience). IL-17 staining was performed using a specific antibody (512334, BioLegend). Data were acquired on a FACSCanto II (Becton Dickinson) with DIVA software, and analysis of the data was performed using FlowJo.

### Flow cytometric analysis

For analysis of extracellular markers, cells were stained with Zombie Fixable Viability dye (77184, BioLegend) and unlabelled anti-CD16/32 (101320, BioLegend) to block nonspecific staining in FACS buffer containing PBS with 0.1% BSA and 2 mM EDTA for 15 min in the dark at 4°C. Cells were washed and labelled with fluorochrome conjugated antibodies against cell surface markers in FACS buffer for 30 min in the dark at 4°C. Cells were washed twice and incubated in fixation solution (BD) for 15 min at room temperature. Cells were washed and re-suspended in PBS prior to acquisition.

For intracellular proteins, cells were stained as above then fixed and permeabilised using the FoxP3/transcription factor staining buffer set (00-5523-00, eBioscience) according to the instructions provided. Cells were then washed and stained for intracellular markers in permeabilisation buffer for 45 min in the dark at 4°C. Prior to acquisition, cells were washed twice with permeabilisation buffer and resuspended in PBS. To detect T cell cytokines, cells were stimulated with 20ng/mL phorbol 12-myristate 13-acetate (P8139, Sigma), 0.4 μM ionomycin (407950, Sigma), and 1.25 μg/ml brefeldin A (0215902705, MP Biomedicals) for 4 hours prior to staining.

Cells were stained with the following antibodies: anti-human CD4 (45-0049-42, eBioscience), anti-human IL-17A (512334, BioLegend), anti- mouse CD4 (12-0041-82, eBioscience), anti-mouse CD25 (102035, BioLegend), anti-Foxp3 (25-5773-82, eBioscience), anti-IFN-γ (48-7311-82, eBioscience), anti-IL-17A (506928, BioLegend), anti-T-bet (45-5825-82, eBioscience), CD69 (164202, BioLegend), ICOS (Inducible Co-Stimulator, 107705 or 107711, BioLegend), anti PD-1 (Programmed Death-1, CD279; 135218, BioLegend), anti-MHCII (107636, BioLegend), anti-F4/80 (EMR1; 123120, BioLegend), anti-CD38 (102730, BioLegend), anti-CD206 (141727, BioLegend). Samples were acquired on a Canto II or LSR II or LSR-Fortessa (BD) and analysed using FlowJo Software.

### Quantification of cytokines

Enzyme-linked immunosorbent assays (ELISAs) were performed on the clarified supernatants using kits from Invitrogen (TNF-α- 88-7324 and IFN-γ- 88-8314-88) according to the manufacturer’s instructions. The plates were read using a SPECTROstar Nano microplate reader (BMG LABTECH) at a wavelength of 450 nm. Cytokine secretion by colon organ cultures was measured using the Meso Scale Discovery (MSD) platform according to the manufacturer’s instructions. A 3-(4, 5-dimethylthiazol-2-yl)-2,5-diphenyltetrazolium bromide (MTT) assay (Sigma) was performed on cells after the collection of medium necessary for analysis to determine cell viability; after incubation with the reagent (3 h) cells were solubilized with 10% w/v SDS overnight at 37degC then analysed on a plate reader; absorbance was measured at 570 nm, with measurement at 690 nm used for background absorbance.

### Determination of kynurenine concentration

Cell culture medium was centrifuged to pellet debris and the clarified supernatant was mixed in a 2:1 ratio with trichloroacetic acid and mixed. The sample was then centrifuged at 500 g for 20min to pellet precipitated proteins. The supernatant was mixed in a 1:1 ratio with Ehrlich’s reagent [20 mg P-dimethylbenzaldehyde/mL acetic acid]. The absorbance was read at 496 nm and compared to a standard of kynurenine concentrations.

### qPCR

RNA extraction was completed according to manufacturer’s instructions (RNeasy Mini Kit, *Qiagen*). A total of 500 ng of RNA was reverse transcribed to cDNA according to the manufacturer’s instructions (High Capacity cDNA Reverse Transcription Kit, *Applied Biosystems*) and diluted to 120 µL. Expression of target genes was determined using TaqMan gene expression assays (*ThermoFischer Scientific*) in duplicate using 2.4 μL of cDNA. Gene expression was calculated relative to the housekeeper gene (*HPRT1*) using the δδCT approximation method.

### Reagents

2-Heptyl-3-hydroxy-4(1H)-quinolone (PQS, 94398, Sigma), Ferrous Sulphate Heptahydrate (F8263, Sigma), γ-Aminobutyric acid (GABA, A2129, Sigma), 4-Hydroxy-2,5-dimethyl-3(2H)-furanone (W317403, Sigma).

### Statistics

Comparisons of two datasets were made by unpaired two-tailed *t* tests with P<0.05 defined as significant using Prism 7 or Instat software (Graphpad). Comparisons of multiple datasets were made by one-way ANOVA followed by the Bonferroni *ad hoc* multiple comparison test for selected datasets. A P value <0.05 was considered statistically significant.

## Results

### PQS differentially affects *in vivo* models of inflammation

As quorum sensing molecules may interact with multiple physiological processes, including the immune response, we tested PQS in three different models of inflammatory disease – CIA, AIA and DSS-induced colitis.

CIA is regarded as the most appropriate model of human rheumatoid arthritis (RA), as it requires cell-mediated and humoral immunity to reproduce the features of human RA. These include joint inflammation and auto-antibodies, and in suitably susceptible mice does not require any external trigger beyond immunisation (36).

AIA has a significant involvement of adaptive immunity due to injection of the sensitizing antigen (mBSA) directly into the knee joint after immunisation (37). The acute DSS model is a chemically-initiated, partly neutrophil-driven, inflammatory model that reproduces many aspects of human ulcerative colitis, including damage to the gut epithelium and infiltration by neutrophils and monocytes; this in turn causes diarrhoea and weight loss until cessation of DSS administration, whereupon the disease usually resolves (38). These models involve overlapping but distinct immune processes from which we sought to deconvolute the potential effects of PQS.

### PQS exacerbates symptoms of CIA

In view of the reports that PQS can modify the production or action of inflammatory mediators, we have examined its effects in the mouse CIA model. PQS could be administered to mice at 10 mg/kg with no change in animal behaviour or locomotion, food intake, body weight or social interactions compared to control mice treated with vehicle alone. This dose was therefore administered to a group of control mice and a group immunised with collagen type II in CFA (28, 39). Following collagen administration the animals were monitored daily for clinical signs of paw swelling (see Methods) and altered gait indicative of arthritis and its associated discomfort. PQS was administered daily from the time of onset of clinical symptoms.

**Figure 1:**
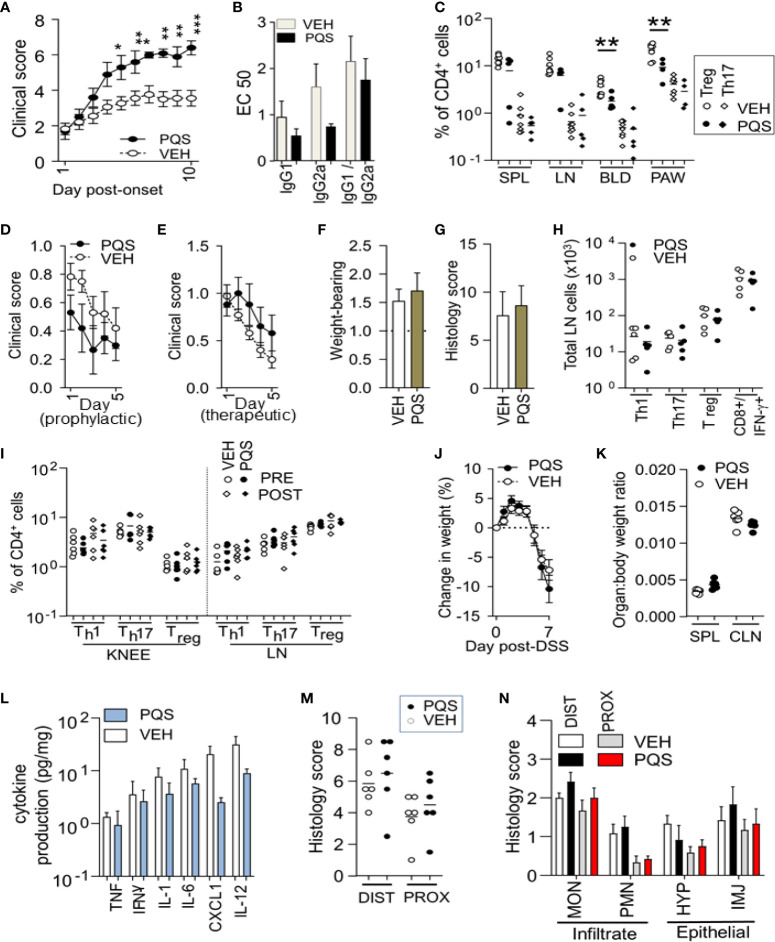
The effect of PQS differs between *in vivo* models of disease. PQS modulation of disease activity in CIA **(A–C)**, AIA **(D–I)** and DSS **(J–N)**. **(A)**: Clinical severity from the onset of paw swelling (day 1) to the termination of the experiment (day 10); **(B)** Anti-collagen humoral responses for IgG1 and IgG2a antibodies and their ratio IgG2a:IgG1; titres were measured by a dilution assay and the results expressed as EC50; *p<0.05, **p<0.01, ***p<0.001 (n = 8 vehicle, n = 5 PQS); **(C)** Analysis of Th17^+^ or CD4^+^FoxP3^+^ Treg cells in affected paws (PAW), lymph nodes (LN) spleens (SPL) and blood (BLD); **P<0.01 (vehicle n = 8; PQS n = 5); **(D)** score (change in knee width) after immunisation and **(E)** after intra-articular injection; n = 5; **(F)** Weight-bearing and **(G)** histological scores of joint damage in AIA mice; **(H)** Th1, Th17 or Treg cells in the inguinal lymph nodes of vehicle or PQS-treated animals; n = 6; **(I)** CD4+ Th1, Th17 and FoxP3+ T cells in the arthritic knee or lymph nodes of mice with AIA; n = 6; **(J)** Dextran sulphate induced changes in body weight, n = 6; **(K)** Ratio of the weights of spleen or colon with total body weight; n = 6; **(L)** Log cytokine production by *ex vivo* colon cultures for TNF, IFN-γ, IL-1β, IL-6, CXCL1 and IL-12 (n = 6); **(M)** Overall histological assessment of the distal (DIST) and proximal (PROX) colon; **(N)** Analysis of infiltrating monocytes (MON) or granulocytes (PMN), and the hyperplasia (HYP) or injury (INJ) of intestinal epithelial cells (n = 6).

PQS treatment produced an increase in the clinical severity of the induced arthritis, with the severity rising steadily from the first dose of PQS and becoming statistically significant from the fifth day (**Figure 1A**). Symptom intensity was maintained until the experiment was terminated on day 10 after symptom onset. There was an accompanying trend of reduction in the mean plasma levels of IgG1 and IgG2a, suggesting a possible generalised suppression of immune system function (**Figure 1B**) but this trend did not reach statistical significance and, importantly, there was no change in the IgG2a:IgG1 ratio.

At termination of these experiments, the arthritic paws and corresponding controls were removed for analysis, along with blood, lymph nodes, and spleen for flow cytometric analysis of T cell populations (**Figure 1C**). The number of Th17 cells in these tissues was quantified as a fraction of the total number of CD4+ cells observed, with no differences seen between the number of Th17 cells in vehicle and PQS treated mice. In contrast there were very significant reductions in the proportion of CD4+ cells expressing FoxP3+ (Treg cells) in the paws and blood. The spleen and lymph nodes exhibited strong trends towards PQS inhibition.

### AIA is not affected by PQS

AIA generates arthritic symptoms triggered by a specific extraneous antigen (mBSA) administered locally into the test limb after prior immunisation, rather than directly inducing an autoimmune response to a joint-derived antigen (collagen) as in the CIA model. PQS showed a tendency to reduce symptoms in the AIA model when administered from the time of immunisation until intra-articular injection (**Figure 1D**), but tended to increase symptoms when administered only after intra-articular administration of mBSA (**Figure 1E**) although neither trend was statistically significant. The lack of effect was supported by the absence of any change in weight-bearing (**Figure 1F**) or the histological severity of arthritis (**Figure 1G**). In this model, no significant changes were observed in the numbers of Th1, Th17, Treg cells or IL-17+ CD8+ cells in the lymph nodes (**Figure 1H**). Similarly, there were no differences in the proportions of Th1, Th17 or Treg cell numbers in the lymph nodes or symptomatic knee joints (**Figure 1I**).

### PQS does not influence the Dextran-induced model of Ulcerative Colitis

PQS was administered to mice in the DSS model of colitis. There were no significant changes in the total body weight (**Figure 1J**) or weight of the spleen or colon as a fraction of body weight (**Figure 1K**). When cultured for 48 h, colon specimens released cytokines into the medium, with a clear, consistent trend for PQS to reduce the levels of TNF, IFN-γ, IL-1β, IL-6, CXCL1 (KC/GRO) and IL-6 (**Figure 1L**), although none reached statistical significance.

Similarly, analysis of the histological data revealed no differences between the overall histological scores for the distal colon or proximal colon (**Figure 1M**) or the individual comparisons of different leucocyte populations classified as mononuclear infiltrate, polymorphonuclear infiltrate, epithelial hyperplasia or epithelial injury cell counts (**Figure 1N**).

### PQS alters the leucocyte balance and cytokine production

**Figure 2:**
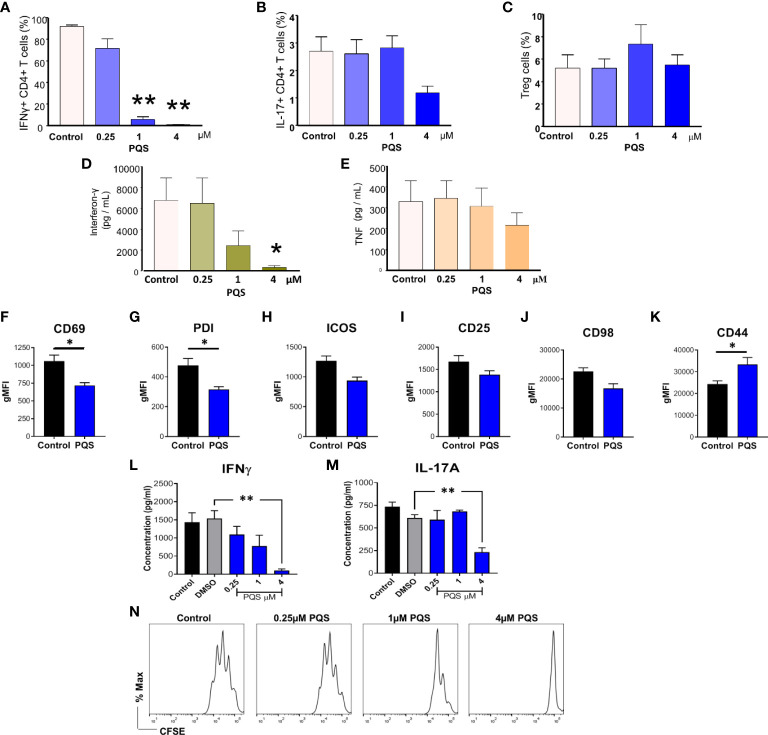
PQS reduces IFN-γ and IL-17 production. T cells isolated from the spleens of mice with CIA, stimulated with anti-CD3 (5 μg/mL) in the absence or presence of PQS for 48 h. Percentage of CD4+ cells expressing **(A)** IFN-γ, IL-17+ and **(C)** FoxP3+ Treg; n = 5. **(D)** IFN-γ and **(E)** TNF secretion from CD4+ splenocytes from CIA mice cultured ex vivo with anti-CD3 in the presence of PQS; n = 4. **(F–K)** Cell surface marker expression on naïve murine T cells stimulated (anti-CD3) in the presence of PQS (n = 4). Secretion of **(L)** IFN-γ and **(M)** IL-17A from cells differentiated to Th1 or Th17, respectively, was inhibited by PQS at 4 μM (n = 3). *p<0.05, **p<0.01, relative to DMSO. **(N)** Representative histogram of CFSE-labelled CD4^+^ cell proliferation indicating inhibition by PQS at 1 and 4 μM. * p<0.05, ** p<0.01, n = 3. (**A-F**: unpaired t test; **G, H**: One way ANOVA and Bonferroni multiple comparison test).

In the light of these results we hypothesized that the exacerbation of CIA caused by PQS might be due to interference with the pro-resolving effects of IFN-γ. Using CIA *ex vivo* antibodies, the inclusion of PQS resulted in a substantial reduction of live, IFN-γ+ cells (**Figure 2A**), whereas the average proportion of IL17+ cells generated was lower but not significantly reduced at 4 μM PQS (**Figure 2B**) and the proportion of Treg cells was unaffected (**Figure 2C**). These changes suggest an increased sensitivity of Th1 cells to PQS compared to other T cell subsets, with lower sensitivity of Th17 and no change on Treg cells. The high potency of PQS activity on Th1 cells was confirmed by a direct assessment of IFN-γ release by anti-CD3 stimulation (5 μg/mL) (**Figure 2D**) where PQS was active at 1 and 4 μM. In comparison, induced release of TNF from the same cells was only partially inhibited, but not significantly even at 4 µM PQS (**Figure 2E**).

### PQS alters the generation and activity of leucocyte populations

Using T cells from naïve mice stimulated by anti-CD3/anti-CD28 for 48 h, PQS at 4 μM reduced the activation of T cells as indicated by a significantly reduced expression of CD69 (**Figure 2F**) and Programmed Death-1 (PD1) (**Figure 2G**). Inducible T cell Co-Stimulator (ICOS) (**Figure 2H**), CD25 (**Figure 2I**) and CD98 (**Figure 2J**) expression were unaffected, but CD44 was increased (**Figure 2K**). PQS at 4 μM almost eliminated the induced secretion of IFN-γ (**Figure 2L**) whereas it had less effect on IL-17A (**Figure 2M**) associated with a reduced overall T cell proliferation observed by FACS analysis (**Figure 2N**).

### PQS alters the balance of T cell differentiation

**Figure 3:**
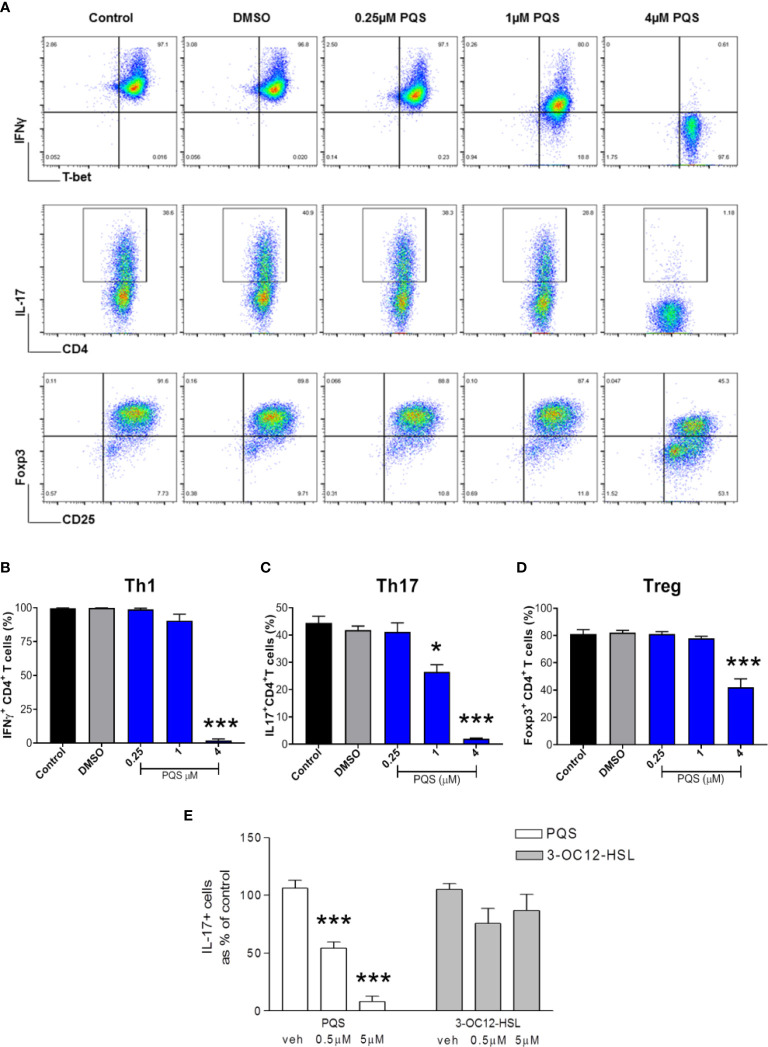
PQS inhibits mouse T cell differentiation. **(A)** Representative FACS plots of mouse leucocytes differentiated to T helper cells in the presence of up to 4 μM PQS. Top, middle and lower rows are Th1, Th17 and Treg-differentiation cultures, respectively. Quantification of **(B)** Th1, **(C)** Th17 and **(D)** Treg cells; *p<0.05, ***p<0.001 (n = 6), **(E)** PQS at 0.5 or 5 μM reduced the generation of IL-17+ T cells in contrast the homoserine lactone 3-OC12-HSL. ***P<0.001 (PQS n=3; 3-OC12-HSL n = 4) (One way ANOVA; Bonferroni *post hoc* test).

Mouse CD4+ cells were differentiated to IFN-γ+ (Th1), IL-17+ (Th17) or FoxP3+ (Treg) cell phenotypes (**Figure 3A**) and stimulated using anti-CD3/CD28 for 4 days in the absence or presence of PQS. The production of Th1 (IFN-γ+) cells was not significantly affected by 1 μM PQS but was fully inhibited at 4 μM (**Figure 3B**). In contrast the generation of Th17 cells was significantly reduced by 1 μM PQS and fully suppressed by 4 μM PQS (**Figure 3C**), while the generation of FoxP3+ Treg cells was reduced only ~50% by 4 μM (**Figure 3D**).

To compare these murine results with human cells, Th17 cells were isolated from human blood. The fraction of these cells present was reduced very significantly after incubation with PQS at 0.5 or 5 μM (**Figure 3E**). These human samples were also used to compare PQS as a representative of the 2A4Q quorum sensor family, and an acyl-homoserine lactone. It was noted that in contrast to the effect of PQS, 3-oxo-dodecanoyl-homoserine lactone (3OC12- HSL) had no significant effect on the proportion of IL17+ cells obtained, indicating a major difference in activity (**Figure 3E**).

### PQS inhibits macrophage activity and polarization

**Figure 4:**
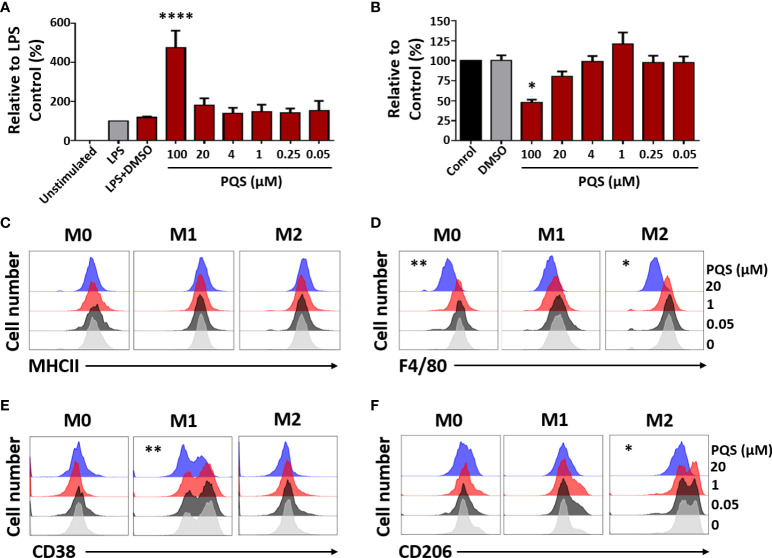
PQS modulates human macrophage polarisation. **(A)** LPS-induced TNF from human monocyte-derived macrophages, in the presence of PQS (0-100 µM); ****P<0.001 relative to LPS plus DMSO (n = 3). **(B)** Cell viability from **(A)** measured by the MTT assay; *P<0.05 relative to DMSO. **(C-F)** M-CSF-differentiated macrophages (M0) were stimulated by M1 (LPS and IFN-γ) or M2 (IL-4 and IL-10) polarizing conditions. The expressions of **(C)** MHC-II, **(D)** F4/80, **(E)** CD38 (M1) and **(F)** CD206 (M2), assessed using FACS, were modulated by PQS (20 μM) (n = 3). For clarity, the FACS results are shown separately for each PQS concentration. The original display and bar chart analysis are illustrated in **Supplementary Figure 1**. *P < 0.05, **P<0.01 relative to DMSO.

Since low concentrations PQS have been reported not to affect TNF release in human cells (40) we examined this possibility in a population of human monocyte-derived macrophages. Here, PQS (up to 10 μM) did not affect TNF release induced by LPS (**Figure 4A**) but at 100 μM, there was an increase in TNF release accompanied by an apparent loss of viability in the MTT assay (**Figure 4B**). This might indicate a general toxicity of PQS at this concentration which could result in the passive efflux of cell contents, including TNF.

Mouse macrophages were induced to differentiate to M1 or M2 phenotypes in the presence of LPS and IFN-γ, or a mixture of IL-4 and IL-10, respectively. FACS analysis of the effects of PQS (0.05, 1 or 20 µM) (**Figures 4C-F**) showed reduced expression of MHC-II (C), the monocyte-derived macrophage (MDM) marker F4/80 (D) (41), the M1 marker CD38 (E) (42) and the M2 marker CD206 (F) (43), indicating inhibition of monocyte differentiation. The original FACS display and an enhanced analysis are shown in Suppl. **Figure 1**.

### PQS reduces the expression of inflammatory mediator genes

**Figure 5:**
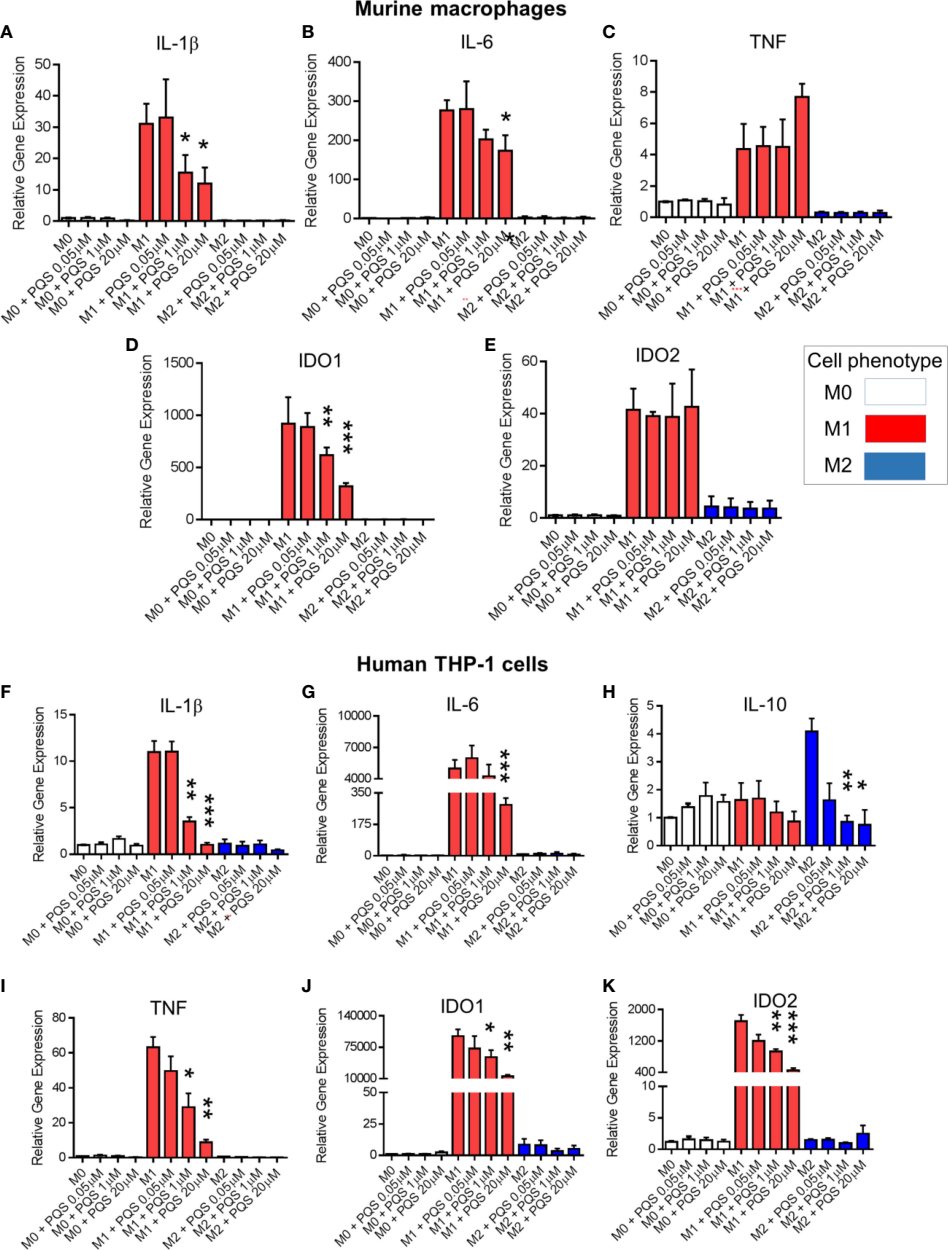
PQS reduces inflammation-associated gene expression in polarized macrophages. In murine bone marrow-derived macrophages the expressions of **(A)** IL-1β, **(B)** IL-6, **(C)** TNF, **(D)** IDO1 and **(E)** IDO2 were measured after polarisation to M0, M1 and M2 macrophages in the presence of PQS (0.5-20 μM). In human THP-1-derived M0, M1 and M2 macrophages cultured in the presence of PQS, the expressions of **(F)** IL-1β, **(G)** IL-6, **(H)** IL-10, **(I)** TNF, **(J)** IDO1 and **(K)** IDO2 were measured; n = 3, *p<0.05, **p<0.01, ***p<0.001.

In murine polarised macrophages, PQS inhibited expression of the genes *Il1b* (**Figure 5A**) and *Il6* (**Figure 5B**), although there was a non-significant tendency to increased expression of *TNF* at higher concentrations of PQS (**Figure 5C**), as observed above in human cells (**Figure 4A**). There was also a differential effect on the major IDO genes, with *Ido1* expression being depressed (**Figure 5D**), but with no change of *Ido2* (**Figure 5E**).

Using human THP-1-derived macrophages, PQS again suppressed expression of IL1B (**Figure 5F**) and IL6 (**Figure 5G**) genes. In cells exposed to M2 phenotypic differentiating conditions there was also an inhibition of IL-10 expression (**Figure 5H**). In M1 polarized cells PQS inhibited TNF gene expression (**Figure 5I**), an effect not seen in primary murine macrophages. A further distinction between cells from the two species was noted with IDO genes, as PQS inhibited expression of both the IDO1 (**Figure 5J**) and IDO2 (**Figure 5K**) genes in the human cells.

**Figure 6:**
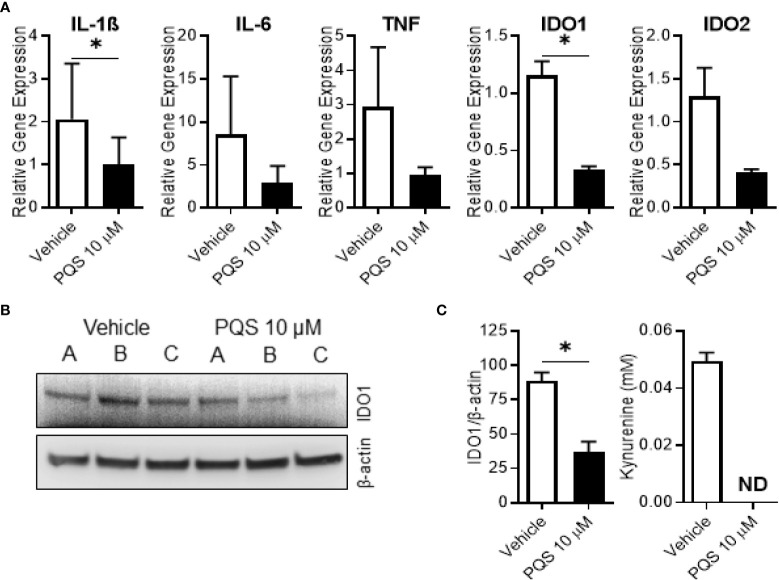
PQS inhibits IDO1 expression in human M1 macrophages. Monocyte-derived macrophages were polarised to an M1 phenotype overnight in the presence or absence of PQS (10µM) to measure gene expression, IDO1 protein expression and IDO1 activity. **(A)** Gene expression of IL1β, IL6, TNF, IDO1 and IDO2; n=3 donors, *p<0.05. **(B)** Western blots of IDO1 and β-actin for 3 independent donors A, B and C. **(C)** (left) -quantification of **(B)**; n=3 donors, *p<0.05; (right) - measurement of kynurenine in the culture medium (ND, not detected); n=3 donors.

To examine its effects on the full range of IDO1 gene, protein and enzyme activity we stimulated human MDMs with LPS in the presence of PQS. PQS reduced the expression of IL-1β and IDO1 genes (**Figure 6A**), with a strong, but not quite significant, effect on IDO2. There was also a significant reduction of IDO1 protein (**Figures 6B, C-left**). As seen in **Figure 6C-right**, PQS abolished the enzyme activity of IDO1, quantified as the lower kynurenine generation.

### Mechanism of action: PQS interacts with iron but not T2 taste receptors

**Figure 7:**
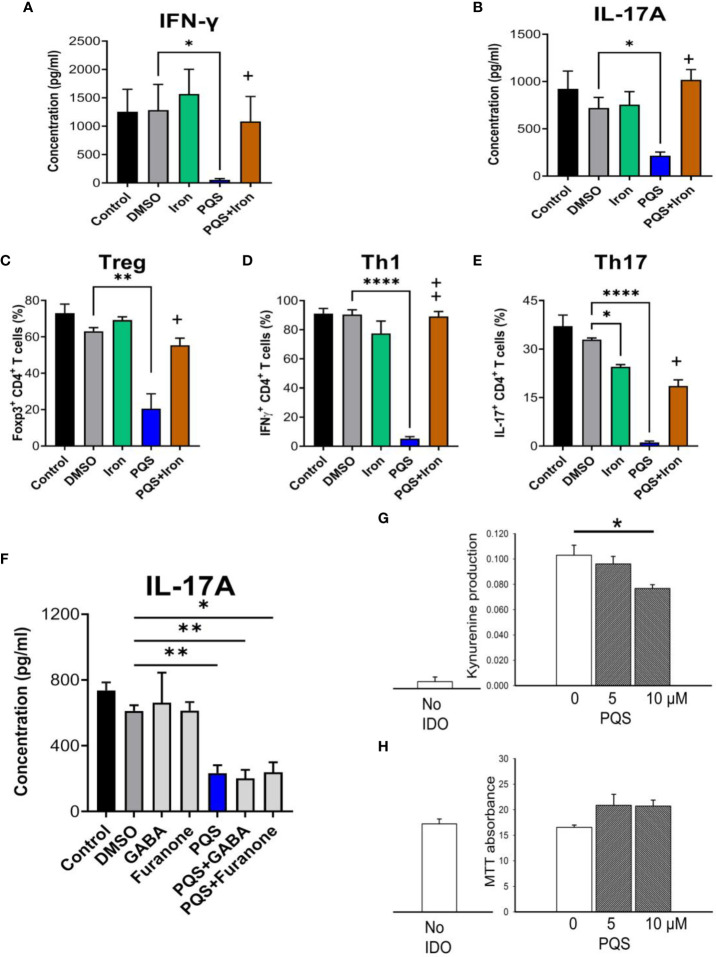
Effects of iron on PQS activity. Inhibition of the release of IFN-γ **(A)** and IL-17A **(B)** in murine T cells by PQS was prevented by FeSO4, which alone did not affect cytokine release. Iron reversed the inhibition by PQS of the *in vitro* differentiation of **(C)** CD4+FoxP3+ Tregs, **(D)** CD4+IFN-γ+ Th1 cells, and **(E)** CD4+IL-17+ Th17 cells; *P<0.05, **P<0.01, ****P<0.001 relative to DMSO; +P<0.05, ++P<0.1 for PQS and iron relative to PQS alone (n = 3). **(F)** GABA and 2,5-dimethyl-furanone had no effect on IL-17A production, and did not prevent the effect of PQS. **(G)** In HEK293 cells over-expressing IDO1, PQS reduced kynurenine in the culture medium with no loss of viability using the MTT assay **(H)**.

Many bacteria chelate iron as a method of limiting the growth and proliferation of competing micro-organisms and PQS enhances cellular responses to iron depletion in some bacteria (44) and in immune system cells of hosts (45, 46). When murine Th1 or Th17 cells were stimulated respectively, the release of cell-specific cytokines was inhibited by PQS at 4μM (**Figures 7A, B**) as noted earlier. The inhibition was fully prevented by including ferrous sulphate in the incubation medium, with complete inhibition of the effects of PQS using 5 μM FeSO4 (**Figures 7A, B**). The generation of FoxP3+CD4+ Treg cells was significantly reduced (**Figure 7C**, as in **Figure 3D** above), with almost complete inhibition of Th1 cells (**Figure 7D**) and Th17 cells (**Figure 7E**), quantified as a fraction of the total CD4+ population. In all cases the inhibitory effects were reversed by the inclusion of iron as ferrous sulphate (**Figures 7A–E**). Comparable results were obtained using iron dextran (data not shown).

An alternative site of action of some QSSMs is the T2 (‘bitter taste’) receptor T2R, which is activated by PQS (47, 48) and blocked by 4-amino-butyric acid (GABA) or 4-Hydroxy-2,5-dimethyl-3(2H)-furanone (2,5-DMF). However, the inhibition of IL-17A production by PQS was not prevented by either of these T2R blockers even at a concentration of 100 μM (**Figure 7F**).

In view of the reversal of PQS activity by iron, the possibility was considered that PQS might interfere with other biological systems relevant to immune function and which employ iron as a critical component. IDO1 is of special interest as it contains an iron-dependent haem moiety and its metabolic oxidation products of tryptophan yields compounds with immune regulatory properties. The enzyme could therefore be a target of PQS during microbial infections. IDO1 enzyme activity was assessed in HEK-293 cells in which the *IDO1* gene had been expressed by transfection. Untransfected HEK-293 cells produced no kynurenine, confirming the absence of endogenous IDO, but HEK293-IDO+ transfected cells generated kynurenine, indicating successful expression of the enzyme (**Figure 7G**). The presence of PQS up to 10 μM produced a statistically significant reduction of kynurenine production consistent with inhibition of IDO1, but without affecting cell viability in the MTT assay (**Figure 7H**).

## Discussion

QSSMs are produced by most prokaryotes and some simple eukaryotes. PQS and related compounds were first described in *Ps. aeruginosa*, a major cause of opportunistic infections with high rates of morbidity and mortality in humans. Although receiving less study, the quinolone derivative PQS is more potent than most homoserine lactone QSSMs (3, 4, 11, 40, 45, 49–57). This high potency may make PQS more relevant in the earliest stages of infectionwhen total bacterial numbers are small. Most of the present experiments used less than 5 μM PQS, while human saliva and blood have levels between 2 μM (49) and around 9 μM during infection (58, 59). The inhibition of cytokine production at low micromolar concentrations and the reduced generation of pro-inflammatory Th1 and Th17 cells seen here may therefore contribute to the infection and invasion of human hosts by bacterial PQS. Higher levels of around 30 μM PQS are produced in culture supernatants of *Ps. aeruginosa* (60, 61) and concentrations achieved by bacterial swarming or biofilms may be over 100 µM *in vivo* (16, 17). This would be relevant to the increased production of TNF seen here at 20 μM PQS and clearly significant at 100 μM. These levels of PQS or other QSSMs may contribute to the development of sepsis (25) and should be recognised as target for anti-bacterial therapeutic strategies in this condition.

### Cytokine production

The production of IFN-γ by Th1 cells is completely suppressed by PQS at a concentration as low as 1 μM. In contrast, low concentrations of PQS failed to alter the secretion of TNF by human macrophages as observed previously (40), but with confirmation of the reported increase in TNF release at higher concentrations of 25 μM or more. Compared with the inhibition of Th17 and Treg cell differentiation, this demonstrates the selectivity of PQS for certain cell populations and cytokine production, supported by our data on human T cells activated by TCR and co-stimulatory anti-CD28. Cytokine selectivity has been reported on other cell types such as bone marrow-derived dendritic cells where, at low concentrations, PQS inhibited LPS-evoked IL-12 production with no change in IL-10 production (11, 40, 49). The inhibitory effects of PQS on cell production of IL-2, IL-6 and IL-12 also depend on the cell type and the nature of any activating stimulus (62). The release of TNF was said to be inhibited (in LPS-activated macrophages) (18, 19, 63, 64), unaffected (in human monocytes) (40), or enhanced at PQS concentrations of 25 μM or above (40). Using similar human primary monocyte-derived macrophages, our results are in agreement with the latter studies, but there may be differences in the behaviour of mouse cells and cultured lines, showing no suppression of TNF release at low PQS concentrations, but increases at 100 μM.

The main populations of T lymphocytes studied here, Th1, Th17 and Treg cells, are critical components of host immune systems against invading micro-organisms and endogenous tumour cells. PQS has differential effects on sub-populations of mouse and human leucocytes, including the suppression of IL-17 expression. The production of several cytokines and metalloproteinases is regulated in part by IL-17, so that sources producing it have become a focus of attention in several inflammatory disorders. Indeed we and others have shown that IL-17, generated primarily from Th17 effector cells, plays a significant role in inflammatory disorders (65, 66). IL-17 is also a potent chemoattractant for monocytes and neutrophils, contributing to their rapid accumulation at sites of infection or tissue damage and probably responsible for some of the pro-inflammatory activity of neutrophils (67, 68). Although less potent, PQS also depressed Treg differentiation, but it is now recognised that immune status is often dependent on the relative amounts of these two populations, and overall pro-inflammatory polarization is assessed using the Th17/Treg ratio (26, 69).

An unexpected outcome of the study was the marked difference between the changes in T cell numbers observed *in vitro* using cells removed from CIA mice and exposed to PQS, and cells isolated from blood, spleen, lymph nodes and inflamed paws of CIA mice treated with PQS. While the balance between Treg and Th17 cell numbers is a key factor in several autoimmune disorders (70, 71), their interdependent differentiation is complex and presents difficulties of interpretation. IL-6 is among the main factors converting FoxP3^+^ Treg cells to the Th17 phenotype and is often found in high concentration in the synovium of patients with arthritis. In addition, a range of materials can influence the Th17/Treg cell balance including small nucleic acid fragments such as miR-448 (72) and miR-302 (73), bile acids (74), hydroxychloroquine (75), chemokine receptor CCR7 (76) and activation of TLRs, particularly TLR4 (77). In addition, a major force driving the Treg/Th17 balance is IDO activity (78, 79) which is determined partly by activated AHRs (80, 81) and can be modulated by STAT3 (82) and Hypoxia Inducible Factor-1α and ATP levels (83). The latter is consistent with the promotion of Treg differentiation by hypoxia which is often a feature of inflamed tissues *in vivo* (84) and may be involved in the increased Th17/Treg ratio induced by tissue injury (85). The complication introduced by ‘reverse signalling’ by IDO1 may also be a factor influencing T cell balance (86).

Since these and many other intracellular or endocrinological factors may be operative *in vivo* but not *in vitro*, the changes in T cell populations observed in the two environments and experimental conditions, are likely to differ. Each *in vivo* model is likely to induce a different immunological signature, of which the Th17/Treg balance is only one and that will be influenced by a range of factors including those noted above. We suggest that the present results reinforce this view: data obtained *in vitro* may not accurately reflect changes occurring *in vivo*, and experimental data should only be used for comparative purposes if they are from the same set of *in vitro* paradigms, or from the same *in vivo* model.

### PQS exacerbation of inflammation

From the present study it is clear that PQS reduces the production of Th1, interferon-γ producing cells and Th17 cells *in vitro* but, paradoxically, it exacerbates the symptoms of CIA. The most likely resolution of this may lie in the absence of a significant change in the numbers of those pro-inflammatory cells *in vivo*, together with reduced numbers of Treg cells in the blood and inflamed paws of CIA mice, yielding a net pro-inflammatory balance of cell populations. PQS produced by infecting micro-organisms might therefore contribute to periodic exacerbations (‘flares’) of autoimmune disease symptoms in human patients. If that is so, an antagonist of PQS or an inhibitor of its synthesis would be of significant clinical value.

Despite the ability of PQS to exacerbate the symptoms of CIA, it had no comparable effect in AIA or DSS-colitis. This suggests that the effects of PQS are seen primarily in disorders with an autoimmune component, such as CIA, rather than in conditions triggered by local, directly acting inflammatory stimuli. This, in turn, would be consistent with the view that the CIA model is a more clinically relevant model for understanding autoimmune disorders and, in particular, for the development of novel therapies.

### Mechanism of action: PQS activity depends on iron but not T2 receptors

Iron balance plays a major role in microbial cell proliferation, invasiveness and biofilm formation to protect against host attack (62, 87–96). Iron is also needed for virulence factor production by bacteria (91, 97–100). These roles for iron extend to the host immune system, with the maintenance and regulation of CD4+ T cell populations (101), especially since iron chelation is likely to inhibit host leucocyte proliferation (45, 46, 102–105). PQS can exacerbate the effects of iron depletion on competing bacteria (44). In addition, PQS promotes the production of the iron complexing siderophores pyocyanin and pyoverdin (62, 106–109) which enhance the removal of free iron. Thus, removing the available sources of iron is a potential approach to antimicrobial therapy (95, 110), but would encourage the cytokine inhibitory effects of PQS. The high potency of iron in reversing PQS effects indicates that this is a significant factor in QS function and further emphasises that there must be a fine balance in iron regulation. Hence a complex network of compounds and pathways exists to maintain levels of iron which not only allow normal metabolic activities of the producing cells, but which can be varied as part of the microbial QS strategy to control cell proliferation and immune competence (46, 102) and the activity of the host innate immune system to control an infection.

These considerations are likely to be clinically relevant. Patients suffering from transition metal deficiency are more susceptible to infection (111–113) but since one of the early responses of the innate immune system to infection is to sequester iron into complexes with transferrin or ferritin (114, 115), a positive feedback may be established which would exacerbate the effects of PQS and related virulence factors. As noted earlier, this situation could lead to, or contribute to, the development of sepsis (25) with the additional concern that prolonged inflammation is a driver of carcinogenesis. High dose antibiotics for the control of sepsis might be usefully complemented by inhibitors of PQS synthesis or promoters of its catabolism.

### PQS acts partly by interfering with IDO and the kynurenine pathway

The kynurenine pathway of tryptophan oxidative catabolism in mammals is the pathway responsible for metabolising 95% of non-protein tryptophan (116–120). Tryptophan depletion by IDO activity, together with its generation of kynurenine and metabolites, exert a variety of actions on the immune system (27, 120–127). A possible relationship between PQS and the kynurenine pathway is especially relevant in view of the effect of PQS on the CIA model of arthritis described here. This model has become established as the preferred system for researching the mechanisms of human RA. Induction of the kynurenine pathway, or the administration of kynurenine itself, reduces the symptoms of CIA, whereas the deletion or pharmacological inhibition of IDO1 exacerbates the disorder (65), suggesting a possible aetiological and therapeutic relevance of the pathway. Similarly, expanding the numbers of Tregs, as would result from kynurenine inducing FoxP3 expression, ameliorates the symptoms (128).

To our knowledge, this is the first report of PQS effects on IDO expression. Even at the low, biologically relevant concentrations employed here PQS reduced the expression of IDO1 in primary monocyte-derived macrophages. This would reduce the tolerogenic activity of those antigen presenting cells, facilitating microbial invasion while inhibiting tumorigenesis. Unusually, PQS also inhibited IDO2 expression. This enzyme has a more limited tissue distribution than the ubiquitous IDO1, but has overlapping tolerogenic activity (129). In addition, it has recently been found to exhibit non-enzymic actions which at present are not fully understood but which are likely to impact on immune cell function (130).

IDO-1 is a heme-containing protein which, accordingly, requires the presence of iron (131–134). As a result, compounds which chelate iron can be efficient inhibitors of IDO1 activity (135–140) and IDO1 inhibitors have been developed for their iron complexation properties (136–138, 141–148) and their resulting anti-cancer activity  (126, 149). The inhibitory effect of nitric oxide on IDO activity may also involve complex formation with the haem iron of IDO1 (141).

The exacerbation of CIA by PQS may therefore have been at least partly attributable to iron chelation and a resulting reduction of IDO1 activity. This explanation was strongly supported by the statistically significant reduction of kynurenine generation by PQS with an approximately 25% inhibition by PQS at 10 μM. It will be interesting to probe this result in greater detail, since it may have been limited by several complicating factors. The cells used here were the human cell line HEK-293 which do not express IDO1 constitutively but into which IDO1 had been transfected. This may mean that essential co-factors required for naturally expressed IDO1 were missing or present in inadequate concentrations. It is also possible, for example, that the enzyme is not expressed with the tertiary structure or spatial orientation which allows PQS to access the iron atom optimally compared to cells *in situ*. Finally, the inhibitory effect of PQS might be much larger in cells activated by compounds which normally induce and activate IDO1 such as interferon-γ or IL-1β.

It should be emphasised that there are alternative factors which may be relevant to the exacerbation of inflammation. For example, the production of IL-10 by macrophages polarised to the M2 phenotype was substantially inhibited by PQS. Since IL-10 has a range of anti-inflammatory actions, including the suppression of inflammatory cytokine expression (150–152), its loss may contribute to the exacerbation of arthritic symptoms independently of - or possibly synergistically with - a suppression of IDO. However, the inhibition of Th1 cells by PQS may make this possibility less likely.

### Wider implications for inter-kingdom communication

PQS is synthesised from anthranilic acid in the bacterial shikimate pathway for tryptophan synthesis (153). Since anthranilate is also a component of the kynurenine pathway it may be a key compound in the communication between bacteria and mammalian hosts, a phenomenon known as ‘inter-kingdom communication’ (154). Modifying tryptophan metabolism is known to affect the production of PQS and related QSSMs (155).

In the presence of inflammation, levels of anthranilate are increased in some disorders, while 3HAA levels are reduced, normalising with treatment (100, 156, 157). Since 3HAA is an effective inhibitor of pro-inflammatory Th1 cells (158, 159) changes in the anthranilate/3HAA ratio resulting from bacterial anthranilate synthesis and catabolism may impact on key elements of host immune function. The small RNAs, PrrF1 and PrrF2, involved in iron regulation and virulence factor production (160), also influence anthranilate degradation (161). Overall, the combined impact of bacteria on anthranilate or 3HAA on PQS production could exert a significant influence on host immunity.

## Data availability statement

The raw data supporting the conclusions of this article will be made available by the authors, without undue reservation.

## Ethics statement

All procedures were approved by the Animal Welfare Ethical Review Board and were undertaken in accordance with personal and project licences issued by the UK Home Office under the UK Animals (Scientific Procedures) Act, 1986.

## Author contributions

TS and RW initiated the project and TS drafted the report. JO, Y-SH, FC, EP, and LT performed the experiments. JO, Y-SH, FC, RW, and TS contributed to the planning of experiments, discussion and interpretation of results. All co-authors JO, Y-SH, FC, EP, LT, LD, RW, and TS read, edited modified and approved the final manuscript.

## Funding

This study was supported by LAB282 (LAB282-2017) and Epsom Medical Research (EMR-2019OX).

## Conflict of interest

The authors declare that the research was conducted in the absence of any commercial or financial relationships that could be construed as a potential conflict of interest.

## Publisher’s note

All claims expressed in this article are solely those of the authors and do not necessarily represent those of their affiliated organizations, or those of the publisher, the editors and the reviewers. Any product that may be evaluated in this article, or claim that may be made by its manufacturer, is not guaranteed or endorsed by the publisher.
