# Supplementary material for: Modulation of immune cell function, IDO expression and kynurenine production by the quorum sensor 2-heptyl-3-hydroxy-4-quinolone (PQS)
Source: Front Immunol. 2022 Oct 28;13:1001956. doi: 10.3389/fimmu.2022.1001956 (PMC9650388; doi:10.3389/fimmu.2022.1001956)
Supplement: Supplementary file 1 [file Image_1.pdf]

Supplementary Figure 1

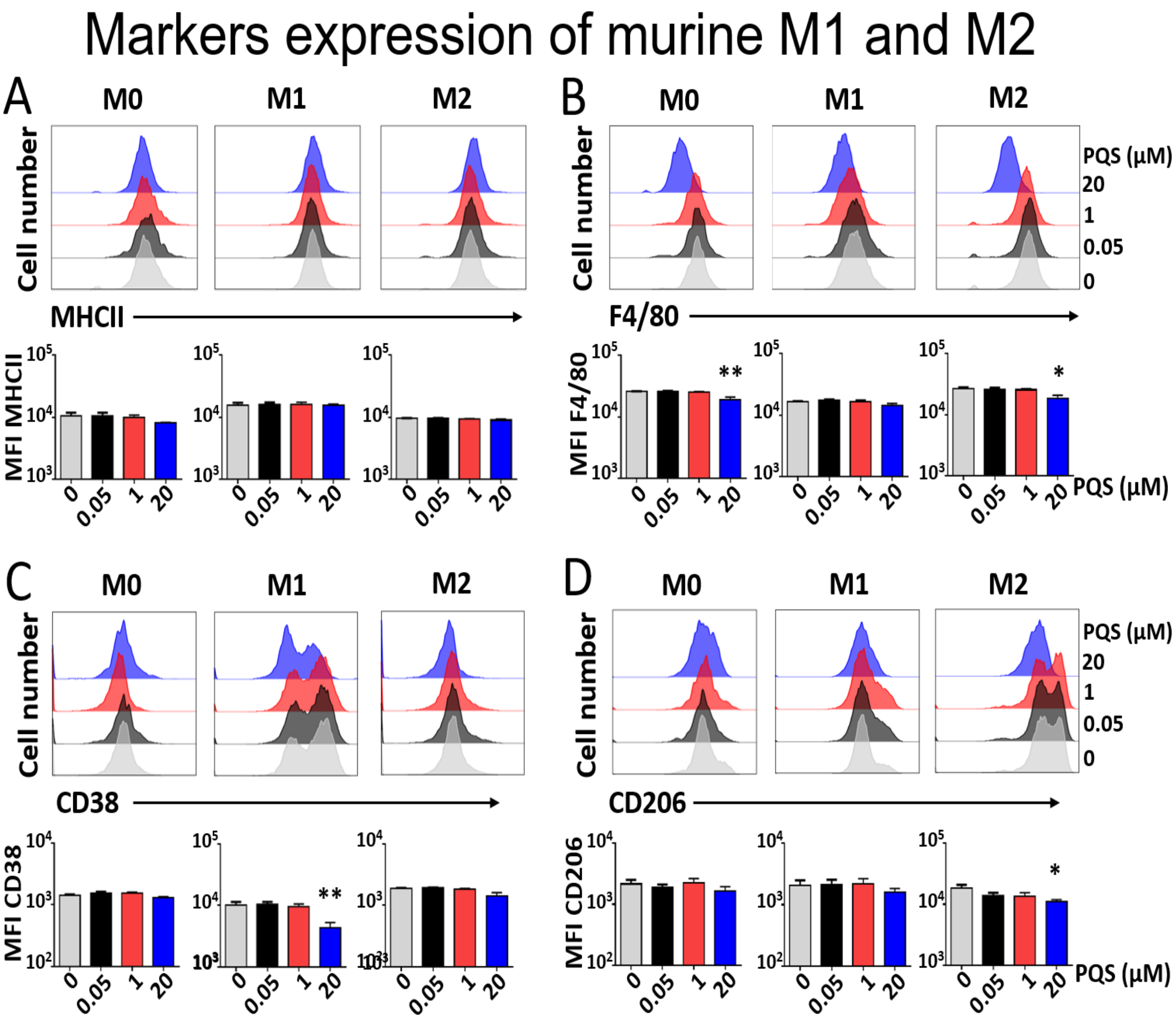

Supplementary Figure 1

(A) The original FACS display of the effects of PQS on macrophage differentiation illustrated in Figure 4 C-F. (B) The separation of the graphical components of the analysis is repeated here from Figure 4, accompanied by a statistical analysis shown in the associated bar charts. *P<0.05, **P<0.01 (one-way ANOVA, Bonferroni multiple comparison test relative to drug-free control).
